# External validation of clinical severity scores to guide referral of paediatric acute respiratory infections in resource-limited primary care settings

**DOI:** 10.1038/s41598-023-45746-4

**Published:** 2023-11-03

**Authors:** Arjun Chandna, Lazaro Mwandigha, Constantinos Koshiaris, Direk Limmathurotsakul, Francois Nosten, Yoel Lubell, Rafael Perera-Salazar, Claudia Turner, Paul Turner

**Affiliations:** 1 Cambodia Oxford Medical Research Unit, Angkor Hospital for Children, Siem Reap, Cambodia; 2 Centre for Tropical Medicine and Global Health, University of Oxford, Oxford, UK; 3 Department of Primary Care Health Sciences, University of Oxford, Oxford, UK; 4 Mahidol-Oxford Tropical Medicine Research Unit, Faculty of Tropical Medicine, Mahidol University, Bangkok, Thailand; 5 Department of Tropical Hygiene, Faculty of Tropical Medicine, Mahidol University, Bangkok, Thailand; 6 Shoklo Malaria Research Unit, Faculty of Tropical Medicine, Mahidol University, Mae Sot, Thailand

**Keywords:** Infectious diseases, Respiratory tract diseases

## Abstract

Accurate and reliable guidelines for referral of children from resource-limited primary care settings are lacking. We identified three practicable paediatric severity scores (the Liverpool quick Sequential Organ Failure Assessment (LqSOFA), the quick Pediatric Logistic Organ Dysfunction-2, and the modified Systemic Inflammatory Response Syndrome) and externally validated their performance in young children presenting with acute respiratory infections (ARIs) to a primary care clinic located within a refugee camp on the Thailand-Myanmar border. This secondary analysis of data from a longitudinal birth cohort study consisted of 3010 ARI presentations in children aged ≤ 24 months. The primary outcome was receipt of supplemental oxygen. We externally validated the discrimination, calibration, and net-benefit of the scores, and quantified gains in performance that might be expected if they were deployed as simple clinical prediction models, and updated to include nutritional status and respiratory distress. 104/3,010 (3.5%) presentations met the primary outcome. The LqSOFA score demonstrated the best discrimination (AUC 0.84; 95% CI 0.79–0.89) and achieved a sensitivity and specificity > 0.80. Converting the scores into clinical prediction models improved performance, resulting in ~ 20% fewer unnecessary referrals and ~ 30–50% fewer children incorrectly managed in the community. The LqSOFA score is a promising triage tool for young children presenting with ARIs in resource-limited primary care settings. Where feasible, deploying the score as a simple clinical prediction model might enable more accurate and nuanced risk stratification, increasing applicability across a wider range of contexts.

## Introduction

Acute respiratory infections (ARIs) are the leading reason for unscheduled childhood medical consultations worldwide^1,2^. Primary care workers function as gatekeepers to the formal health system, aiming to distinguish the minority of ARIs requiring onward referral from those suitable for community-based care^3^.

In rural regions of many low- and middle-income countries (LMICs) poorly functioning infrastructure, as well as geographic, climatic, socioeconomic, and cultural factors, can complicate referral mechanisms. Particularly in humanitarian and conflict settings referral can entail risks for both patients and providers^4^. Consequently, there can be substantial inter- and intra-health system variation in referral thresholds.

Existing tools to support community healthcare providers in their assessment of unwell children, such as the World Health Organization’s Integrated Management of Childhood Illnesses (IMCI) and Integrated Community Case Management (iCCM) guidelines, recommend certain ‘Danger Signs’ to guide referrals^5,6^. However, these lack sensitivity and specificity, and suffer from considerable interobserver variability^7,8^. A systematic review of paediatric triage tools concluded that none would be reliable in resource-constrained settings and that lack of follow-up data on children managed in the community rendered the validity of existing tools questionable^9^.

In this study we identified paediatric severity scores suitable for use in resource-limited primary care settings and externally validated their ability to guide referral of young children presenting with ARIs^10^. We characterised the improvement in performance that might be expected if the scores were deployed as simple clinical prediction models and updated to include variables relevant to children presenting with ARIs in rural LMIC settings.

## Methods

### Study population

Data were collected during a prospective birth cohort study at a medical clinic for refugees and internally displaced people on the Thailand-Myanmar border^10^. Between September 2007 and September 2008 pregnant women receiving antenatal care at the clinic were invited to participate. Children of consenting women were reviewed at birth and followed-up each month (routine visit) and during any intercurrent illness (illness visit) until 24 months of age. The local circumstances (inability of the population to move freely out of the camp and lack of other medical providers) contributed to low attrition rates and capture of the majority of acute illnesses for which care was sought.

All ARI illness visits were included in this secondary analysis. An ARI was defined as (A) a presentation with rhinorrhoea, nasal congestion, cough, respiratory distress (chest indrawing, nasal flaring, grunting, tracheal tug, and/or head bobbing), stridor, and/or abnormal lung auscultation (crepitations and/or wheeze), and (B) a compatible contemporaneous syndromic diagnosis (rhinitis, croup, bronchiolitis, influenza-like illness, pneumonia, viral infection, and/or wheeze) for children sent home directly from the clinic.

### Identification and shortlisting of scores

Drawing on the results of two recent systematic reviews, we longlisted 16 severity scores that might risk stratify young children presenting from the community with ARIs (Supplementary Table 1)^11,12^. After considering reliability, validity, and feasibility for implementation we excluded eight scores that required specialist equipment and/or laboratory tests unlikely to be practical for the assessment of young children in busy LMIC primary care settings^13–20^. Four others were excluded as ≥ 25% of the constituent variables were unavailable in the primary dataset (Supplementary Table 2)^21–24^. Two of the remaining scores (the quick Sequential Organ Failure Assessment [qSOFA] and the quick Pediatric Logistic Organ Dysfunction-2 [qPELOD-2]) contained blood pressure^25,26^. Hypotension is a late sign in paediatric sepsis and not suitable for early recognition of impending serious illness at the community level^27^. Furthermore, accurate use and maintenance of sphygmomanometers and stethoscopes may not be feasible in resource-limited settings^28^. Recently, Romaine et al. replaced systolic blood pressure (SBP) with alternate signs of circulatory compromise (heart rate and capillary refill time) to develop the Liverpool-qSOFA (LqSOFA) score, and demonstrated superior performance compared to qSOFA in febrile children presenting from the community^29^. Hence, we elected to evaluate the LqSOFA score in preference to qSOFA and to evaluate an adapted qPELOD-2 score (replacing SBP with capillary refill time and assessing mental status using the simpler Alert Voice Pain Unresponsive [AVPU] scale rather than the Glasgow Coma Scale [GCS]). The three scores shortlisted for evaluation were the LqSOFA, qPELOD-2, and modified Systemic Inflammatory Response Syndrome (mSIRS) scores (Table 1)^26,29,30^.

**Table 1:** Shortlisted paediatric severity scores and comparison between original and study populations.

Score	Constituent variables	Population	Outcome
LqSOFA^29^	1. Capillary refill time > 2 s 2. Mental status < alert on AVPU scale 3. Heart rate > age-adjusted threshold 4. Respiratory rate > age-adjusted threshold *Each variable allocated one point to give score of 0–4*	*Derivation*: 1121 febrile children < 16 years attending the ED and requiring a blood test at a specialist paediatric hospital in the United Kingdom *Validation*: 12,241 febrile children < 16 years attending the ED at a specialist paediatric hospital in the United Kingdom	Critical care admission within 48 h of ED attendance *Prevalence*: 4.2% (derivation) and 1.1% (validation)
mSIRS^30^	1. Core temperature > 38.5 °C or < 36 °C 2. Heart rate > or < age-adjusted threshold 3. Respiratory rate > age-adjusted threshold *Each variable allocated one point to give score of 0–3*	*Derivation*: expert consensus (original SIRS score)^18^. *Validation*: 1184 adults > 18 years admitted to a hospital in Sri Lanka with suspected infection	In-hospital mortality, cardiac arrest, or ICU admission (validation) *Prevalence*: 3.6% (validation)
qPELOD-2^26^	1. Mental status < 11 on GCS 2. Heart rate > age-adjusted threshold 3. Blood pressure < age-adjusted threshold *Each variable allocated one point to give score of 0–3*	*Derivation*: 862 children < 18 years admitted to nine European PICUs with suspected infection *Validation*: 545 children < 18 years admitted to a hospital in the Netherlands with suspected bacterial infection^17^	In-PICU mortality (derivation) or PICU admission and/or mortality (validation) *Prevalence*: 7.0% (derivation) and 3.3% (validation)
This study	1. Capillary refill time > 2 s 2. Mental status < alert on AVPU scale 3. Heart rate > or < age-adjusted threshold 4. Respiratory rate > age-adjusted threshold 5. Axillary temperature > 38 °C or < 35.5 °C	3010 ARI presentations from 756 children < 2 years presenting to a primary care clinic on the Thai-Myanmar border	Supplemental oxygen therapy *Prevalence*: 3.5%

*AVPU Alert Voice Pain or Unresponsive, bpm* beats or breaths per minute, *ED* emergency department, *ICU* intensive care unit, *PICU* paediatric intensive care unit.

### Selection of variables for model updating

To update and improve model performance additional predictors relevant for children presenting with ARIs in LMIC primary care settings were considered for inclusion. Nutritional status (weight-for-age z-score [WAZ]) and presence of respiratory distress were selected a priori, after considering resource constraints, reliability, validity, biological plausibility, availability of data in the primary dataset, and sample size (Supplementary Table 3)^28^.

### Data collection

All data were measured by study staff and entered onto structured case report forms. With the exception of anthropometric data, all clinical data were collected at the time of presentation. Core (rectal) temperature was measured for neonates and infants and adjusted to axillary temperature by subtracting 0.5 °C^6^. Mental status was assessed using the AVPU scale. Capillary refill time was measured following the release of gentle pressure on the child’s sternum. For children admitted to the clinic, weight was measured at the time of presentation (seca scale; precision ± 5 g for neonates or ± 50 g after birth). In addition, all children had their mid-upper arm circumference (MUAC), weight, and height measured at each monthly routine visit. For the purposes of these analyses, age-adjusted z-scores (R package: *z scorer*)^31^ were calculated using the closest anthropometric data to the illness visit within the following window periods: height ≤ 28 days; MUAC ≤ 28 days without intervening admission; weight ≤ 14 days without intervening admission. Median time between the index illness visit and each anthropometric measurement is reported. Participants were followed-up each day during admission to the clinic and at monthly routine visits conducted as part of the longitudinal birth cohort study.

### Primary outcome

The primary outcome was receipt of supplemental oxygen at any time during the illness visit. Study staff were unaware which baseline variables were to be used as candidate predictors at the time of ascertaining outcome status. Clinic treatment protocols specified that peripheral oxygen saturation (SpO_2_) must be checked prior to initiation of supplemental oxygen, with therapy only indicated if SpO_2_ was < 90%. Elevation within the camps ranged from 200 to 1000 m and adjustment of SpO_2_ readings for altitude was not required^32^. All staff had either undergone formal nurse training in Myanmar before being displaced to the camp or had undergone a 6-month training programme in the camp (run by Médecins Sans Frontières). All had more than a year’s clinical experience in the camp and were trained on the clinic treatment protocols prior to study commencement.

### Missing data

616 presentations were missing data on one or more candidate predictors (616/3010; 20.5%) with capillary refill time containing the highest proportion of missingness (442/3,010; 14.7%; Supplementary Table 4). Under a missing-at-random assumption (Supplementary Fig. 1; Supplementary Table 5), we used multiple imputation with chained equations (MICE) to deal with missing data (R package: *mice*)^33^. Analyses were done in each of 100 imputed datasets and results pooled. Variables included in the imputation model are reported in Supplementary Table 6.

### Statistical methods

We assessed discrimination and calibration of each score by quantifying the area under the receiver operating characteristic curve (AUC) and plotting the observed proportion of participants that met the primary outcome at each level of a score. We examined predicted classifications at each of the scores’ cut-offs.

Prior to model building we explored the relationship between continuous predictors and the primary outcome using locally-weighted scatterplot smoothing (LOWESS) to identify non-linear patterns^34^. Accordingly, temperature was modelled using restricted cubic splines (R package: *rms*)^35^ with three knots placed at locations based on percentiles (5th and 95th) and recognised physiological thresholds (36 °C)^36,37^. We used logistic regression to derive the models and tested for important interactions using likelihood ratio tests (LRT). Although a number of children presented more than once during the study period, mixed-effects models accounting for repeat presenters failed to converge due to a substantial proportion of children presenting only once (22%; 169/756), hence random-effects were not modelled. Sensitivity analyses restricting the analysis to one ARI presentation per child indicated that this is unlikely to have had an impact on the findings (Supplementary Table 7). All predictors were prespecified and no predictor selection was performed during model development. Internal validation was performed using 100 bootstrap samples with replacement and optimism-adjusted discrimination and calibration reported (R package: *rms*)^35^.

Finally, the models were updated by including respiratory distress and WAZ as additional candidate predictors. Penalised (lasso) logistic regression was used for model updating, variable selection, and shrinkage to minimise overfitting (R package: *glmnet*)^38^. A sensitivity analysis confirmed that median imputation grouped by outcome status produced similar results to MICE and hence to avoid conflicts in variable selection across multiply imputed datasets we used this approach to address missing data for model updating (Supplementary Table 8). We assessed discrimination and calibration of the updated models, examined predicted classifications at clinically-relevant referral thresholds, and compared their clinical utility (net-benefit) to the best-performing points-based severity score using decision curve analysis (R package: *dcurves*)^39^. A sensitivity analysis was performed excluding children who were hypoxaemic at the time of presentation.

All analyses were done in R, version 4.0.2^40^.

### Sample size

No formal sample size calculation for external validation of the existing severity scores was performed. All available data were used to maximise power and generalisability. Of the 3010 eligible ARI presentations, 104 met the primary outcome, ensuring sufficient outcome events for a robust external validation^41^. For derivation and updating of the clinical prediction models we followed the methods of Riley et al. and assumed a conservative R^2^ Nagelkerke of 0.15^42^. At an outcome prevalence of 3.5% (104/3010) we estimated that up to 13 candidate predictors (events per parameter [EPP] = 8) could be used to build the prediction models whilst minimising the risk of overfitting (R package: *pmsampsize*)^43^.

### Ethics and reporting

Ethical approvals were provided by the Mahidol University Ethics Committee (TMEC 21-023) and Oxford Tropical Research Ethics Committee (OxTREC 511-21). Informed consent was obtained from the legal guardians of all participants. The study is reported in accordance with the Transparent Reporting of a multivariable prediction model for Individual Prognosis Or Diagnosis (TRIPOD) guidelines (Supplementary Table 9)^44^.

## Results

From September 2007 to September 2008, 999 pregnant women were enrolled, with 965 children born into the cohort. Amongst 4061 acute illness presentations, 3064 were for ARIs. Fifty-four ARI presentations were excluded as information on oxygen therapy was not available in the study database, leaving 3010 presentations from 756 individual children for the primary analysis (Supplementary Fig. 2).

Baseline characteristics of the cohort are summarised (Table 2; Supplementary Table 10). The majority of children were managed in the community (72.3%; 2175/3010). Median length of stay for the 835 admissions was 3 days (IQR 2–4 days). One hundred and four (3.5%; 104/3010) presentations received supplemental oxygen during their illness visit (met the primary outcome), with those with signs of respiratory distress, age-adjusted tachycardia and/or tachypnoea, lower baseline SpO_2_, prolonged capillary refill times, altered mental status, and lower WAZ more likely to require supplemental oxygen (*p* < 0.001 to 0.014; Table 2). There was one death: a child who was admitted and received supplemental oxygen.

**Table 2:** Baseline characteristics of the cohort stratified by primary outcome status.

Characteristic	Overall N = 3010^1^	Supplemental oxygen		*p* value^2^
		No N = 2906^1^	Yes N = 104^1^	
**Demographics**				
Age (months)	8.1 (3.7, 13.7)	8.2 (3.8, 13.8)	7.3 (3.4, 12.7)	0.40
Male sex	1592/3010 (53%)	1541/2906 (53%)	51/104 (49%)	0.40
**Birth history**				
Gestation (weeks)^e^	39.1 (38.1, 40.0)	39.2 (38.2, 40.0)	38.4 (37.3, 39.7)	0.001
Birthweight (kg)^e^	2.9 (2.6, 3.2)	2.9 (2.6, 3.2)	2.6 (2.0, 3.0)	< 0.001
**Previous medical history**				
Number of previous illness visits	3.0 (2.0, 6.0)	3.0 (2.0, 6.0)	4.0 (2.0, 9.0)	0.043
Time since last illness visit (days)	29.0 (3.0, 81.0)	31.0 (3.0, 82.0)	11.0 (2.0, 36.5)	< 0.001
Number of previous ARI visits	3.0 (2.0, 5.0)	3.0 (2.0, 5.0)	3.5 (2.0, 8.0)	0.006
Known comorbidity^e^	53/3000 (1.8%)	39/2898 (1.3%)	14/102 (14%)	< 0.001
**History of current illness**				
Duration of symptoms (days)^e^	3.0 (2.0, 5.0)	3.0 (2.0, 5.0)	3.0 (2.0, 5.0)	0.30
Antibiotics prior to presentation	145/3010 (4.8%)	125/2906 (4.3%)	20/104 (19%)	< 0.001
Family member unwell^e^	287/3000 (9.6%)	276/2898 (9.5%)	11/102 (11%)	0.70
**Presenting symptoms and signs**				
Fever^e^	1958/3005 (65%)	1885/2901 (65%)	73/104 (70%)	0.30
Cough	2767/3010 (92%)	2667/2906 (92%)	100/104 (96%)	0.11
Runny nose^e^	2565/3008 (85%)	2491/2904 (86%)	74/104 (71%)	< 0.001
Noisy breathing^e^	447/3004 (15%)	430/2901 (15%)	17/103 (17%)	0.60
Stridor^e^	6/3009 (0.2%)	6/2905 (0.2%)	0/104 (0%)	> 0.90
Respiratory distress^ae^	508/3009 (17%)	416/2905 (14%)	92/104 (88%)	< 0.001
*Head bobbing*^e^	52/3009 (1.7%)	27/2905 (0.9%)	25/104 (24%)	< 0.001
*Tracheal tug*^e^	134/3009 (4.5%)	96/2905 (3.3%)	38/104 (37%)	< 0.001
*Grunting*^e^	26/3009 (0.9%)	11/2905 (0.4%)	15/104 (14%)	< 0.001
*Chest indrawing*^e^	493/3009 (16%)	402/2905 (14%)	91/104 (88%)	< 0.001
Abnormal lung auscultation^be^	1455/2951 (49%)	1372/2852 (48%)	83/99 (84%)	< 0.001
*Crepitations*^e^	1158/2941 (39%)	1085/2844 (38%)	73/97 (75%)	< 0.001
*Wheeze*^e^	794/2931 (27%)	751/2833 (27%)	43/98 (44%)	< 0.001
Dehydration^e^	127/3003 (4.2%)	121/2899 (4.2%)	6/104 (5.8%)	0.40
Pale, mottled or cyanosed^e^	107/2960 (3.6%)	91/2862 (3.2%)	16/98 (16%)	< 0.001
**Vital signs**				
Heart rate (bpm)^e^				
*Neonate*	140.0 (132.0, 150.0)	140.0 (132.0, 148.0)	150.0 (140.0, 165.0)	0.014
*Infant*	138.0 (128.0, 144.0)	136.0 (128.0, 144.0)	147.0 (136.5, 154.0)	< 0.001
*Child*	128.0 (120.0, 140.0)	128.0 (120.0, 140.0)	140.0 (127.5, 149.0)	0.002
Respiratory rate (bpm)^e^				
*Neonate*	48.0 (45.0, 56.0)	48.0 (44.2, 54.0)	64.5 (54.0, 77.0)	0.008
*Infant*	48.0 (42.0, 56.0)	48.0 (42.0, 56.0)	58.0 (54.0, 66.0)	< 0.001
*Child*	45.0 (38.0, 52.0)	44.0 (38.0, 52.0)	57.0 (46.5, 62.0)	< 0.001
Axillary temperature (°C)^ce^	36.6 (36.0, 37.5)	36.6 (36.0, 37.4)	36.8 (36.2, 37.8)	0.040
Oxygen saturation (%)^e^	95.0 (93.0, 96.0)	95.0 (93.0, 96.0)	88.0 (85.0, 93.0)	< 0.001
Capillary refill time > 2 s^e^	36/2568 (1.4%)	27/2476 (1.1%)	9/92 (9.8%)	< 0.001
Not alert^e^	372/2973 (13%)	306/2875 (11%)	66/98 (67%)	< 0.001
**Anthropometrics**				
Weight-for-length z-score (WLZ)^d,e^	0.0 (− 0.8, 0.8)	0.0 (− 0.8, 0.8)	− 0.5 (− 1.8, 0.7)	< 0.001
Weight-for-age z-score (WAZ)^d,e^	− 0.9 (− 1.6, − 0.2)	− 0.9 (− 1.6, − 0.2)	− 1.9 (− 3.4, − 0.8)	< 0.001
MUAC-for-age z-score (MAZ)^d,e^	0.2 (− 0.4, 0.8)	0.2 (− 0.4, 0.8)	− 0.7 (− 1.9, 0.6)	< 0.001
Length-for-age z-score (LAZ)^d,e^	− 1.5 (− 2.3, − 0.7)	− 1.4 (− 2.2, − 0.7)	− 2.4 (− 3.4, − 1.4)	< 0.001

^a^Respiratory distress defined as head bobbing, tracheal tug, grunting, and/or chest indrawing.

^b^Abnormal chest auscultation defined as crepitations and/or wheeze.

^c^Rectal temperature converted to axillary temperature for neonates and infants.

^d^Median interval between anthropometric measurement and index illness presentation: length = 8 days (IQR 4–12 days); MUAC = 9 days (IQR 4–13 days); weight = 4 days (IQR 0–10 days).

^e^Missing data: gestation = 5; birthweight = 14; comorbidity = 10; symptom duration = 21; unwell family member = 10; fever = 5; runny nose = 2; noisy breathing = 6; stridor = 1; respiratory distress = 1; head bobbing = 1; tracheal tug = 1; grunting = 1; chest indrawing = 1; abnormal lung auscultation = 59; lung crepitations = 69; wheeze = 79; dehydration = 7; colour = 50; heart rate = 9; respiratory rate = 8; temperature = 3; oxygen saturation = 1645; capillary refill time = 442; mental status = 37; WLZ = 158; WAZ = 147; MAZ = 682; LAZ = 14.

^1^Median (IQR); n/N (%).

^2^Wilcoxon rank sum test; Pearson's Chi-squared test; Fisher's exact test.

### LqSOFA and qPELOD-2 scores outperform the mSIRS score for risk stratification of ARIs

**Figure 1:**
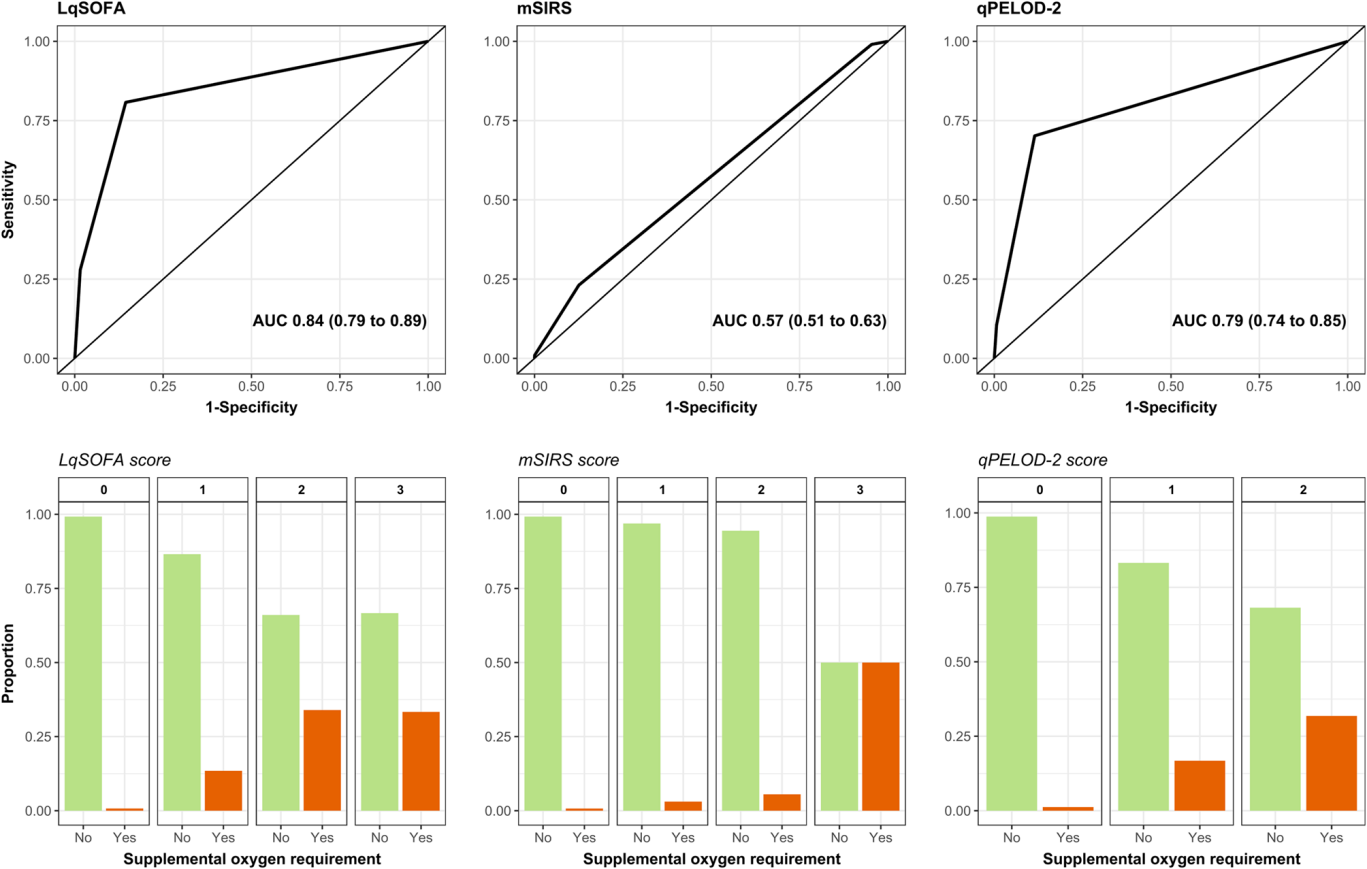
Discrimination of the LqSOFA, mSIRS, and qPELOD-2 severity scores. Receiver operating characteristic curve (ROC) for one imputed dataset shown. Variability in ROCs across multiply imputed datasets shown in Supplementary Fig. 2. Pooled AUC reported (100 bootstrap samples). Bar plots showing observed proportion of ARI presentations which developed an oxygen requirement at each level of a score using full case analysis: LqSOFA = 2525 presentations (81 met primary outcome); mSIRS = 2992 presentations (99 met primary outcome); qPELOD-2 = 2531 presentations (83 met primary outcome).

Discrimination and calibration of the LqSOFA (AUC = 0.84; 95% confidence interval [CI] = 0.79 to 0.89) and qPELOD-2 (AUC = 0.79; 95% CI = 0.74 to 0.84) scores were considerably better than the mSIRS score (AUC = 0.57; 95% CI = 0.51 to 0.63; Fig. 1; Supplementary Table 11; Supplementary Fig. 3). At a cut-off of ≥ 1 the LqSOFA score demonstrated a sensitivity of 0.80 (95% CI = 0.72 to 0.89) and specificity of 0.86 (95% CI = 0.85 to 0.88); neither the mSIRS nor qPELOD-2 scores achieved a sensitivity and specificity > 0.70 at any cut-off (Table 3).

**Table 3:** Predicted classifications of the severity scores.

Cut off	Sensitivity (95% CI)	Specificity (95% CI)	Negative predictive value (95% CI)	Positive predictive value (95% CI)	Negative likelihood ratio (95% CI)	Positive likelihood ratio (95% CI)	Cases referred (%)	Cases managed in community (%)	Ratio of incorrect to correct referrals	Ratio of correct to incorrect cases managed in community
**LqSOFA**										
≥ 1	0.80 (0.72–0.89)	0.86 (0.85–0.88)	0.99 (0.99–1.00)	0.16 (0.13–0.20)	0.23 (0.15–0.36)	5.89 (5.08–6.82)	407 (16.1%)	2118 (83.9%)	5 to 1	131 to 1
≥ 2	0.23 (0.14–0.33)	0.98 (0.98–0.99)	0.98 (0.97–0.98)	0.34 (0.22–0.46)	0.78 (0.69–0.88)	15.49 (9.33–25.72)	68 (2.7%)	2457 (97.3%)	3 to 1	39 to 1
≥ 3	0.01 (-0.01–0.04)	1.00 (1.00–1.00)	0.97 (0.96–0.98)	0.33 (-0.20–0.87)	0.99 (0.96–1.01)	15.09 (1.38–164.69)	1 (< 0.01%)	2524 (> 99.9%)	0 to 1	31 to 1
**mSIRS**										
≥ 1	0.99 (0.97–1.00)	0.05 (0.04–0.05)	0.99 (0.98–1.01)	0.03 (0.03–0.04)	0.22 (0.03–1.54)	1.04 (1.02–1.06)	2846 (95.1%)	146 (4.9%)	28 to 1	145 to 1
≥ 2	0.22 (0.14–0.30)	0.88 (0.86–0.89)	0.97 (0.96–0.98)	0.06 (0.03–0.08)	0.89 (0.80–0.99)	1.79 (1.22–2.61)	369 (12.3%)	2623 (87.7%)	16 to 1	33 to 1
≥ 3	0.01 (-0.01– 0.03)	1.00 (1.00–1.00)	0.97 (0.96–0.97)	0.50 (-0.19–1.19)	0.99 (0.97–1.01)	29.22 (1.84–463.84)	1 (< 0.1%)	2991 (> 99.9%)	0 to 1	30 to 1
**qPELOD-2**										
≥ 1	0.68 (0.57–0.78)	0.90 (0.88–0.91)	0.99 (0.98–0.99)	0.18 (0.14–0.22)	0.36 (0.27–0.50)	6.40 (5.30–7.73)	301 (11.9%)	2230 (88.1%)	4 to 1	82 to 1
≥ 2	0.08 (0.03–0.14)	0.99 (0.99–1.00)	0.97 (0.96–0.98)	0.32 (0.12–0.51)	0.92 (0.86–0.98)	13.76 (5.77–32.86)	31 (1.2%)	2500 (98.8%)	3 to 1	32 to 1

Classifications calculated using full-case analysis: LqSOFA = 2525 presentations (81 met primary outcome); mSIRS = 2992 presentations (99 met primary outcome); qPELOD-2 = 2531 presentations (83 met primary outcome).

### Improved performance of clinical severity scores when deployed as clinical prediction models

**Figure 2:**
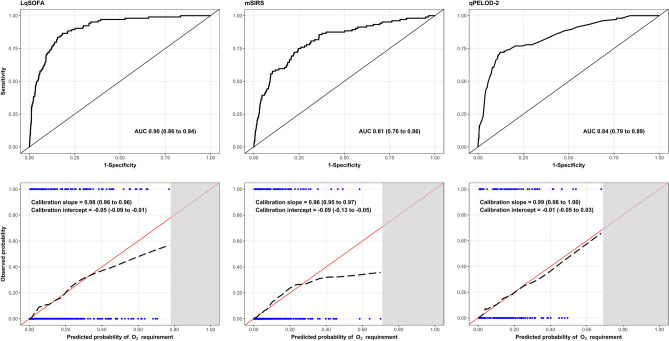
Discrimination and calibration of the LqSOFA, mSIRS, and qPELOD-2 models. Receiver operating characteristic curve (ROC) and calibration slope for one imputed dataset shown. Variability in ROCs and calibration slopes across multiply imputed datasets shown in Supplementary Fig. 5. Pooled optimism-adjusted AUCs and calibration slopes reported (100 bootstrap samples). On calibration plots, red line indicates perfect calibration; black dashed line indicates calibration slope for that particular model; blue rug plots indicate distribution of predicted risks for participants who did (top) and did not (bottom) meet the primary outcome.

Relationships between continuous predictors and the primary outcome are illustrated (Supplementary Fig. 4). There was no evidence of interaction between heart rate (LRT = 2.09; *p* = 0.35) or respiratory rate (LRT = 0.77; *p* = 0.68) and age. Optimism-adjusted discrimination of the three models ranged from 0.81 to 0.90, with the LqSOFA model appearing most promising (AUC = 0.90; 95% CI = 0.86 to 0.94; Fig. 2; Supplementary Fig. 5). Calibration of the qPELOD-2 model was good. The LqSOFA and mSIRS models overestimated risk at higher predicted probabilities.

**Figure 3:**
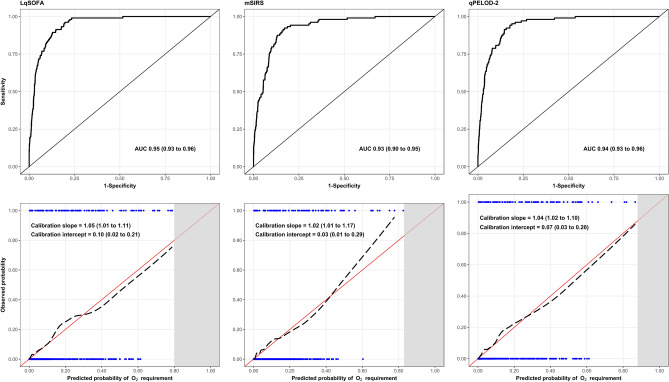
Discrimination and calibration of the updated LqSOFA, mSIRS, and qPELOD-2 models. On calibration plots, red line indicates perfect calibration; black dashed line indicates calibration slope for that particular model; blue rug plots indicate distribution of predicted risks for participants who did (top) and did not (bottom) meet the primary outcome.

Discrimination of all three updated models containing respiratory distress and WAZ improved (AUCs = 0.93 to 0.95). Notably, improvements were more substantial for the qPELOD-2 and mSIRS models, compared to the LqSOFA model, which already had comparably high discrimination prior to inclusion of the additional variables. Calibration of the updated LqSOFA and qPELOD-2 models was good, whereas the updated mSIRS model underestimated risk at higher predicted probabilities (Fig. 3). The full models are reported in Supplementary Table 12.

### Promising clinical utility of the LqSOFA and qPELOD-2 models to guide referrals from primary care

**Figure 4:**
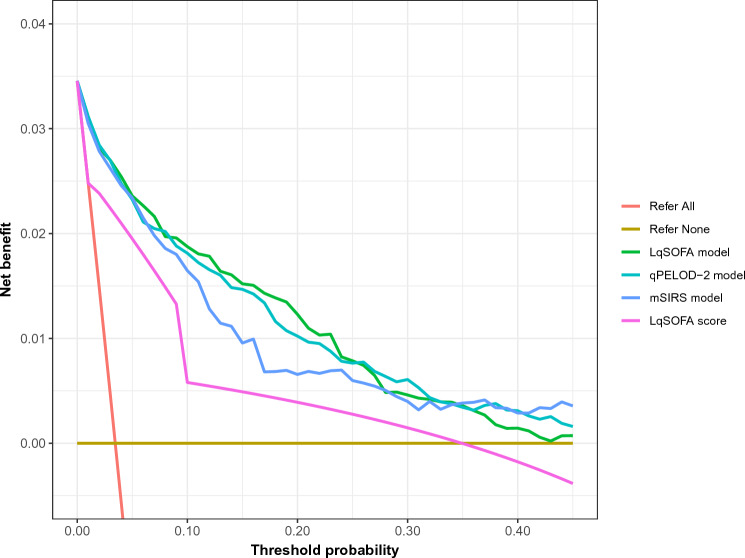
Decision curve analysis of the updated LqSOFA, mSIRS, and qPELOD-2 models. The net benefit of the updated models (green [LqSOFA], turquoise [qPELOD-2], and blue [mSIRS] lines) and original LqSOFA score (pink line), are compared to a “refer-all” (red line) and “refer-none” (brown line) approach. A threshold probability of 5% indicates a management strategy whereby any child with a ≥ 5% probability of requiring oxygen is referred (i.e., a scenario where the value of one correct referral is equivalent to 19 incorrect referrals or a NNR of 20). *NNR* number needed to refer.

We recognised that the relative value of correct and incorrect referrals is highly context-dependent, reflecting resource availability, practicalities of referral, and capacity for follow-up. Decision curve analyses accounting for differing circumstances suggest that the updated models could provide greater utility (net-benefit) compared to the best points-based score (the LqSOFA score), with the LqSOFA and qPELOD-2 models appearing most promising over a wide range of plausible referral thresholds (Fig. 4).

The ability of each updated model to guide referrals at thresholds ranging from 1 to 40% is shown (Table 4). A referral threshold of 5% reflects a strategy whereby any child with a predicted probability of requiring oxygen ≥ 5% is referred. At this cut off, the models would suggest referral in ~ 15% of all presentations, correctly identifying ~ 86 to 87% of children requiring referral, at a cost of also recommending referral in ~ 12 to 13% of children not requiring referral; i.e., a number needed to refer (NNR; the number of children referred to identify one child who would require oxygen) of five. In contrast, at a similar threshold the LqSOFA score using a cut-off ≥ 1 would suggest referral in a similar proportion of presentations but result in a ~ 25% increase in incorrect referrals (a NNR of six) and a ~ 25-30% increase in the number of children incorrectly identified as safe for community-based management (a ratio of correct to incorrect cases managed in the community of 171 to 193:1 vs. 131:1).

**Table 4:** Predicted classifications at different referral thresholds using the updated LqSOFA, qPELOD-2, and mSIRS models.

Model	Sensitivity (95% CI)	Specificity (95% CI)	Negative predictive value (95% CI)	Positive predictive value (95% CI)	Negative likelihood ratio (95% CI)	Positive likelihood ratio (95% CI)	Cases referred (%)	Cases managed in community (%)	Ratio of incorrect to correct referrals	Ratio of correct to incorrect cases managed in community
**Referral threshold = 1%**										
LqSOFA	0.97 (0.93–1.00)	0.78 (0.73–0.82)	1.00 (1.00–1.00)	0.14 (0.11–0.17)	0.04 (0.00–0.09)	4.48 (3.65–5.57)	723 (24.0%)	2287 (76.0%)	6 to 1	571 to 1
qPELOD-2	0.96 (0.93–0.99)	0.79 (0.75–0.83)	1.00 (1.00–1.00)	0.14 (0.12–0.17)	0.05 (0.01–0.10)	4.55 (3.97–5.87)	715 (23.8%)	2295 (76.2%)	6 to 1	573 to 1
mSIRS	0.94 (0.90–0.98)	0.78 (0.65–0.84)	1.00 (1.00–1.00)	0.13 (0.10–0.18)	0.08 (0.04–0.14)	4.36 (3.00–6.21)	714 (24.5%)	2296 (75.5%)	6 to 1	382 to 1
**Referral threshold = 5%**										
LqSOFA	0.87 (0.78–0.93)	0.88 (0.86–0.91)	0.99 (0.99–1.00)	0.21 (0.18–0.25)	0.15 (0.09–0.25)	7.40 (6.22–9.50)	423 (14.1%)	2587 (85.9%)	4 to 1	171 to 1
qPELOD-2	0.87 (0.78–0.93)	0.87 (0.85–0.91)	0.99 (0.99–1.00)	0.20 (0.16–0.23)	0.15 (0.08–0.25)	6.79 (5.96–8.98)	468 (15.5%)	2542 (84.5%)	4 to 1	180 to 1
mSIRS	0.86 (0.77–0.93)	0.87 (0.85–0.89)	0.99 (0.99–1.00)	0.19 (0.16–0.22)	0.16 (0.08–0.26)	6.55 (5.80–7.74)	470 (15.6%)	2540 (84.4%)	4 to 1	193 to 1
**Referral threshold = 10%**										
LqSOFA	0.75 (0.66–0.83)	0.93 (0.91–0.95)	0.99 (0.99–0.99)	0.29 (0.24–0.36)	0.26 (0.18–0.37)	11.76 (9.04–16.80)	270 (9.0%)	2740 (91.0%)	2 to 1	101 to 1
qPELOD-2	0.73 (0.61–0.82)	0.93 (0.90–0.95)	0.99 (0.99–0.99)	0.29 (0.23–0.37)	0.29 (0.20–0.41)	11.57 (8.21–17.02)	264 (8.8%)	2764 (91.2%)	2 to 1	94 to 1
mSIRS	0.76 (0.63–0.86)	0.91 (0.88–0.93)	0.99 (0.99–0.99)	0.23 (0.19–0.27)	0.27 (0.16–0.41)	8.22 (6.83–10.18)	344 (11.4%)	2666 (88.6%)	3 to 1	110 to 1
**Referral threshold = 20%**										
LqSOFA	0.59 (0.45–0.69)	0.97 (0.96–0.97)	0.99 (0.98–0.99)	0.39 (0.32–0.45)	0.42 (0.32–0.56)	17.82 (13.83–23.17)	161 (5.3%)	2849 (94.7%)	2 to 1	65 to 1
qPELOD-2	0.56 (0.41–0.65)	0.97 (0.96–0.97)	0.98 (0.98–0.99)	0.37 (0.30–0.44)	0.46 (0.36–0.60)	16.81 (12.98–22.87)	153 (5.1%)	2857 (94.9%)	2 to 1	57 to 1
mSIRS	0.49 (0.37–0.61)	0.96 (0.95–0.97)	0.98 (0.98–0.99)	0.31 (0.26–0.38)	0.53 (0.40–0.65)	12.97 (9.85–19.44)	165 (5.5%)	2845 (94.5%)	2 to 1	50 to 1
**Referral threshold = 40%**										
LqSOFA	0.28 (0.16–0.41)	0.99 (0.98–1.00)	0.97 (0.97–0.98)	0.49 (0.36–0.62)	0.73 (0.60–0.85)	27.50 (17.38–56.63)	62 (2.1%)	2948 (97.9%)	1 to 1	36 to 1
qPELOD-2	0.28 (0.13–0.41)	0.99 (0.98–0.99)	0.97 (0.97–0.98)	0.49 (0.35–0.59)	0.73 (0.59–0.87)	27.90 (16.46–47.56)	62 (2.1%)	2948 (97.9%)	1 to 1	39 to 1
mSIRS	0.21 (0.09–0.35)	1.00 (0.99–1.00)	0.97 (0.97–0.98)	0.61 (0.47–0.90)	0.80 (0.66–0.91)	Inf	20 (0.7%)	2990 (99.3%)	1 to 1	34 to 1

A referral threshold of 5% reflects a management strategy whereby any child with a predicted probability of requiring oxygen ≥ 5% is referred.

### Sensitivity analysis

The WHO recommend that pulse oximetry should be universally available at first-level health facilities^6,45^. Although many barriers exist to realising this laudable goal, to account for the fact that in such contexts a severity score would not be required to guide referral for children who are already hypoxaemic at the time of presentation, we performed a sensitivity analysis excluding attendances with SpO_2_ < 90% at presentation. Discrimination remained comparable but clinical utility of the models reduced slightly, with higher NNRs at the lowest referral thresholds (Supplementary Tables 13 and 14).

## Discussion

We report the external validation of three pre-existing severity scores amongst young children presenting with ARIs to a medical clinic on the Thailand-Myanmar border. Unlike other studies which investigated the scores’ prognostic accuracy in hospital settings^17,25^, we evaluated their performance at the community level and demonstrate that the LqSOFA and qPELOD-2 scores could support early recognition of children requiring referral or closer follow-up in primary care settings with limited resources. In keeping with previous literature, we found that the mSIRS score was poorly discriminative, not well calibrated, and led to substantial misclassification^17^.

An LqSOFA score ≥ 1 yielded a sensitivity and specificity > 80%. Encouragingly, this is remarkably consistent with the performance reported in the original LqSOFA development study and may reflect similarities in the use-case (febrile children presenting from the community) and severity of the cohorts (outcome prevalence 1.1% vs. 3.5%; admission rate 12.1% vs. 27.7%), albeit despite obvious demographic differences^29^. In contrast to qPELOD-2, LqSOFA contains age-adjusted tachypnoea, which may have improved performance in children with respiratory illnesses. Furthermore, the performance of LqSOFA (or qSOFA) has been shown to improve outside of the PICU, when predicting more proximal outcomes (e.g., critical care admission rather than mortality), and if the AVPU scale (vs. GCS) is used to assess mental status^46^. These all apply to our cohort.

We demonstrated improvement in performance when the severity scores were deployed as clinical prediction models and when nutritional status and respiratory distress were included as additional predictors. Whilst discrimination of all three updated models was good, the AUC is a summary measure of model performance and does not necessarily reflect clinical utility^47–49^. Decision curve analyses illustrate the superiority of the LqSOFA and qPELOD-2 models compared with the mSIRS model across a range of clinically-relevant referral thresholds.

With growing access to smartphones there may be contexts where the increased accuracy afforded by a clinical prediction model outweighs the simplicity and practicality of points-based scoring systems. At a 5% referral threshold, the updated LqSOFA model identified a similar proportion of presentations for referral as the LqSOFA score at a cut-off of ≥ 1 (14.1% vs. 16.1%), however use of the model would have resulted in ~ 20% fewer incorrect referrals and a ~ 30% decrease in the number of presentations incorrectly recommended for community-based management. In addition to greater accuracy, prediction models permit more nuanced evaluation of risk; referral thresholds can be adjusted to the needs of an individual patient and/or health system and this flexibility may be particularly impactful in the heterogeneous environments commonplace in many LMIC primary care contexts. For example, in locations where community follow-up is feasible (e.g., via a telephone call or return clinic visit) and/or referral carries great cost (to the patient or system), a higher referral threshold (lower NNR) may be acceptable, compared with settings where safety-netting is impractical and/or access to secondary care is less challenging.

We followed the latest guidelines in prediction model building and used bootstrap internal validation, penalised regression, placed knots at predefined locations, and limited the number of candidate predictors to avoid overfitting the models^42,44,50,51^. Nevertheless, they require validation on new data to assess generalisability and provide a fairer comparison with the pre-existing points-based scores. We have published our full models to encourage independent validation.

As others have highlighted, a limitation of many studies evaluating community-based triage tools in resource-limited settings is the lack of follow-up data for patients categorised as low risk^9^; 72.3% (2175/3010) of our cohort were sent away from the clinic without admission. As acute illness visits were nested within the longitudinal birth cohort, we were able to confirm that 1.4% (30/2083) of presentations sent away from the clinic without admission received supplemental oxygen within the next 28 days, although it is unknown whether this related to the index ARI or a new illness. A sensitivity analysis conservatively classifying these 30 presentations as meeting the primary outcome (i.e., assuming the oxygen therapy related to the index ARI) resulted in a decrease in the sensitivity of all three models (Supplementary Tables 15 and 16). Prospective research with dedicated outpatient follow-up is ongoing to investigate this issue further^52^.

We selected supplemental oxygen therapy as the primary outcome as this reflects a clinically-meaningful endpoint for ARIs and a pragmatic referral threshold for many resource-limited primary care settings. Although confirmed hypoxaemia would have been a more robust endpoint, oxygen saturations prior to starting oxygen therapy were not documented in the study database. However, oxygen was a scarce resource during the study (cylinders were transported in each week from ~ 60 km away) and oxygen therapy was protocolised (only indicated if SpO_2_ < 90%); hence outcome misclassification is less likely with the primary outcome reflecting a reliable surrogate for hypoxaemia rather than a subjective decision by a health worker to provide supplemental oxygen.

For those who met the primary outcome, the time of oxygen initiation was not available in the primary dataset. Although no patient had met the outcome when baseline predictors were measured, some may have done so shortly after. Nevertheless, the sensitivity analysis excluding presentations with baseline SpO_2_ < 90% (the qualifying criterion for supplemental oxygen) produced similar results.

Finally, due to the proportion of children who presented only once, we were unable to include a random-effect term in the models. Although this may have biased findings towards those children who presented more frequently, the sensitivity analyses restricted to a single presentation per child indicate that this is unlikely to be the case.

We externally validated three severity scores that could guide assessment of young children presenting with ARIs in resource-limited primary care settings (particularly where pulse oximetry is not readily available) to identify those in need of referral or closer follow-up. Performance of the LqSOFA score was encouraging and comparable to that in the original derivation setting^29^. Converting the LqSOFA score into a clinical prediction model and including additional variables relevant to resource-constrained LMIC settings improved accuracy and might permit application across a wider range of contexts with differing referral thresholds.

### Supplementary Information

Supplementary Information.

## Data Availability

De-identified, individual participant data from this study will be available to researchers whose proposed purpose of use is approved by the data access committees at the Mahidol-Oxford Tropical Medicine Research Unit. Inquiries or requests for the data may be sent to datasharing@tropmedres.ac.
